# Comparing the Incidence of Propranolol and Esmolol-Related Cardiac Arrest in Patients With Thyroid Storm: A Systematic Literature Review

**DOI:** 10.7759/cureus.44655

**Published:** 2023-09-04

**Authors:** Shadin Afifi, Vineet Suryadevara, Yaman Habab, Alana Hutcheson, Binay K Panjiyar, Gershon G Davydov, Hiba Nashat, Sally Ghali, Safeera Khan

**Affiliations:** 1 Internal Medicine, California Institute of Behavioral Neurosciences and Psychology, Fairfield, USA; 2 Global Clinical Scholar Research Training (GCSRT), Post Graduate Medical Education (PGMEE) at Harvard Medical School, Boston, USA; 3 Internal Medicine, California Institute of Behavioral Neurosciences and Psychology, California, USA; 4 Research, California Institute of Behavioral Neurosciences and Psychology, Fairfield, USA; 5 Internal Medicine, Soroka University Medical Center, Beer Sheva, ISR

**Keywords:** beta blockers, cardiac arrest, cardiogenic shock, esmolol, propranolol, thyroid storm, thyrotoxic crisis

## Abstract

A precarious complication of thyrotoxicosis, or thyroid storm, is the increased risk of cardiomyopathy, which leads to circulatory collapse and cardiopulmonary arrest. It is crucial to promptly identify this condition to prevent significant deterioration of the left ventricular function and cardiogenic shock. This article seeks to examine published research that emphasizes the connection between thyroid storm and beta-blocker usage in relation to cardiogenic collapse and provides management recommendations. The search was performed on September 9, 2022, using PubMed, Science Direct, and Google Scholar libraries. A systematic exploration was carried out using the keywords *Thyroid Storm *AND *cardiogenic Shock *AND cardiac arrest AND beta blocker. The use of beta blockers as part of thyroid storm management was linked to the development of cardiogenic collapse and cardiac arrest. Ultra-short-acting beta-blockers like esmolol were a safer option than propranolol in treating patients with a thyrotoxic storm.

## Introduction and background

A thyrotoxic storm (thyroid crisis) is a rare and possibly fatal endocrine emergency characterized by severe hyperthyroidism symptoms. The incidence of thyrotoxic storms ranges between 0.2 and 0.7 per 100,000 people per year, and hospitalization accounts for 4.8 and 5.6 per 100,000 people per year, as per the national surveys in the United States and Japan in the years between 2003 and 2013 [1]. The male-to-female (M/F) ratio is about 1:3, with an average age of occurrence of 42-43 years [2]. The storm can be triggered by any acute stressful event such as surgery, infection, trauma, parturition, acute iodine load, or abrupt disruption of hyperthyroidism treatment in patients with longstanding thyroid disease [2]. The estimated mortality rate is 10-30% [1], mainly due to hypertension, congestive heart failure, arrhythmia, cardiogenic shock, and multi-organ failure [3].

The clinical manifestations of the thyroid crisis range from thermoregulatory, neurologic, gastro-hepatic, and cardiac dysfunctions to circulatory collapse and shock (Table 1) [4]. Burch and Wartofsky's criteria can guide diagnosis; a score of 45 or above strongly indicates a thyroid storm (Table 2) [5,6].

**Table 1:** Clinical manifestations

History of thyroid disease and precipitating factor	Goitre, thyroid eye disease
Thermoregulatory	Fever (low, moderate, or high), rigor, sweating
Neurologic	lethargy, agitation, delirium, psychosis, seizures, coma
Gastro-hepatic	Diarrhoea, vomiting, abdominal pain, jaundice
Cardiology	Palpitation, atrial fibrillation, pulmonary oedema

**Table 2:** Burch and Wartofsky criteria

Criteria	Points
Temperature	(37.2–37.7) = 5
	(37.8–38.3) = 10
	(38.4–38.8) = 15
	(38.9–39.4) = 20
	(39.4–39.9) = 25
	(≥40.0) = 30
Cardiovascular	Heart rate (HR) (100–109) = 5
	(110–119) = 10
	(120–129) = 15
	(130–139) = 20
	(≥40) = 25
	Atrial fibrillation absent = 0
	Present = 10
Congestive heart failure	Mild = 5
	Moderate = 10
	Sever = 20
Neurological disturbance	Mild (agitation) = 10
	Moderate (delirium, psychosis, extreme lethargy) = 20
	Severe (seizures, coma) = 30
Gastrointestinal symptoms	Moderate (diarrhoea, abdominal pain, N/V) = 10
	Sever (jaundice) = 15
Precipitating events	Absent = 0
	Present = 10
Total	>45 = thyroid storm
	25-45 = impending storm
	<25 = storm unlikely

The treatment consists of supportive therapy with IV fluids, electrolyte replacement, oxygen, and specific treatment with beta-blockers, anti-thyroid medication, potassium iodide or lugol iodine, and hydrocortisone. Second-line therapy can be lithium, dialysis, or plasmapheresis (Table 3) [3].

**Table 3:** Treatment summary BNF: beta blockers [7]

Supportive	IV fluids, electrolytes replacement, oxygen
Beta-blocker [7]	Propranolol, bisoprolol, metoprolol, esmolol.
Anti-thyroid	Propylthiouracil, carbimazole, lugol iodine, potassium iodide, hydrocortisone
Second line	Lithium, colestipol, cholestyramine, dialysis, plasmapheresis, or thyroid surgery

Currently, there are no unified clinical guidelines for treating thyroid storms, and clinicians use the available recommendations and hyperthyroidism treatment guides to treat the storm. Furthermore, no clinical trials were conducted or are underway to help establish treatment standards due to the rarity of the condition and how acute it is [3].

In this systemic review article, we attempted to explore the link between propranolol use in thyroid storm treatment and developing circulatory collapse and cardiac arrest. Additionally, we compared the incidence of cardiac arrest with propranolol and esmolol use to help formulate future guidelines.

## Review

Methodology

This review is reported based on the Preferred Reporting Items for Systemic Reviews and Meta-Analysis (PRISMA) guidelines. All the data were collected from published papers only; therefore, no ethical approval was needed.

*Search Strategy* 

The review question was made using the PICO criteria (population, intervention/condition, control, outcome, time, type of study) to look for relevant literature in databases such as PubMed, including MEDLINE and PMC, Science Direct, and Google Scholar Libraries. The keywords used were thyroid storm, propranolol, esmolol, and cardiac arrest, in different combinations with BOOLEANS 'AND and 'OR.

The following search strategy was used: "Thyroid Crisis/complications"[Mesh] OR "Thyroid Crisis/drug therapy"[Mesh] OR "Thyroid Crisis/mortality"[Mesh] OR "Thyroid Crisis/therapy"[Mesh]) and ("Propranolol/adverse effects"[Mesh] OR "Propranolol/therapeutic use"[Mesh] OR "Propranolol/toxicity"[Mesh]). The keywords used for searching Science Direct and Google Scholar were "Thyroid crisis, propranolol, esmolol, collapse," "storm" OR "crisis," "arrest" OR crisis, "collapse" (Table 4).

**Table 4:** Study selection

Database	Search strategy	Search result	Number of longlisted (after applying filter)	Number of shortlisted (after applying eligibility criteria)
PubMed (+MEDLINE and PMC)	PubMed MeSH: ("Thyroid Crisis/complications"[Mesh] OR "Thyroid Crisis/drug therapy"[Mesh] OR "Thyroid Crisis/mortality"[Mesh] OR "Thyroid Crisis/therapy"[Mesh]) and ("Propranolol/adverse effects"[Mesh] OR "Propranolol/therapeutic use"[Mesh] OR "Propranolol/toxicity"[Mesh])	69	-	2
	PMC: (("thyroid crisis"[MeSH Terms] OR ("thyroid"[All Fields] AND "crisis"[All Fields]) OR "thyroid crisis"[All Fields]) AND ("propranolol"[MeSH Terms] OR "propranolol"[All Fields]) AND ("esmolol"[Supplementary Concept] OR "esmolol"[All Fields]) AND ("heart arrest"[MeSH Terms] OR ("heart"[All Fields] AND "arrest"[All Fields]) OR "heart arrest"[All Fields] OR ("cardiac"[All Fields] AND "arrest"[All Fields]) OR "cardiac arrest"[All Fields])) AND ("2012/10/12"[PDat]: "2022/10/09"[PDat])	16	-	6
Science Direct	Thyroid crisis and propranolol and esmolol	274	Review articles, research articles and case reports = 24	1
Google Scholars	Thyroid crisis and propranolol and esmolol	1650	Thyroid crisis and propranolol and esmolol collapse storm or crisis arrest storm OR crisis "collapse"= 62	2

Inclusion and Exclusion Criteria

PICO models were used to select the studies appropriate for eligibility checks. The inclusion criteria were studies published in the last ten years (between 2012 and 2022), adult population (18 years and above), human studies, and only in English. The exclusion criteria were grey literature, animal studies, paediatric populations, unpublished studies, and papers published before 2012. Moreover, repeated studies or papers that did not pass the quality assessment checklists were excluded from this review.

Quality Assessment

The selected articles were subjected to quality assessment using the JB check tool and the CARE checklist for the case reports and case series. At the same time, the a meaSurement tool to assess systematic reviews (AMSTAR) checklist and PRISMA were utilized to conduct the systemic review. The total number of papers shortlisted for review was nine, as detailed in Tables 5-7.

**Table 5:** JB checklist

Paper	Demographic characteristics	The history described clearly and timely	Description of the presenting clinical condition	Description of diagnostic tests or assessment methods and the results	Description of the intervention(s) or treatment procedure(s)	Description of the post-intervention clinical condition	Description of adverse events (harms) or identification of unanticipated events	Does the case report provide takeaway lessons?
Alahmad et.al. [8]	✅	✅	✅	✅	✅	✅	✅	✅
Lim et al. [9]	✅	✅	✅	✅	✅	✅	✅	✅
Wong et al. [10]	✅	✅	✅	✅	✅	✅	✅	✅
Ali et al. [11]	✅	✅	✅	✅	✅	✅	✅	✅
Cheah et al. [12]	✅	✅	✅	✅	✅	✅	✅	✅
Muhailan et al. [13]	✅	✅	✅	✅	✅	✅	✅	✅
Abukaer et al. [14]	✅	✅	✅	✅	✅	✅	✅	✅
Moraco et al. [15]	✅	✅	✅	✅	✅	✅	✅	✅

**Table 6:** CARE checklist CARE: CAse REports

Papers	Title	Keywords	Abstract	Introduction	Patient info	Clinical findings	Timeline	Diagnostic assessment	Therapeutic intervention	Follow-up and outcomes	Discussion	Patient perspective	Informed consent	% of quality
Alahmad et al. [8]	✅	✅	✅	✅	✅	✅	✅	✅	✅	✅	✅	🔶	🔶	85%
Lim et al. [9]	✅	✅	✅	✅	✅❌	✅	✅	✅	✅	✅❌	✅	🔶	🔶	80%
Wong et al. [10]	✅	✅	✅	✅	✅	✅	✅	✅	✅	✅	✅	✅	✅	100%
Ali et al. [11]	✅	✅	✅	❌	✅	✅	✅	✅	✅	✅	✅	🔶	🔶	90%
Chead et al. [12]	✅	✅	✅	✅	✅	✅	✅	✅	✅	✅	✅	🔶	✅	93.5%
Muahilan et al. [13]	✅	✅	✅	✅	✅❌	✅	✅	✅	✅	❌	✅	🔶	🔶	78%
Abubaker et al. [14]	✅	✅	✅	✅	✅	❌	✅	✅❌	✅	✅❌	✅	🔶	✅	80%
Moraco et al. [15]	✅	✅	✅❌	✅	✅	✅	✅	✅	✅	✅❌	✅	🔶	🔶	84%

**Table 7:** PRISMA checklist PRISMA: Preferred Reporting Items for Systemic Reviews and Meta-Analysis

Paper	Title	Abstract	Introduction	Method	Result	Discussion	Other info	% Quality
Modarresi et al. [16]	✅	✅❌	✅	✅❌	✅❌	✅❌	✅❌	70%

Results

A total of 2,009 papers were identified from all the databases used when using the keywords combined: PubMed, MEDLINE, PMC, Science Direct, and Google Scholar. In PubMed using the MeSH strategy, 85 papers were identified, while in Science Direct, 274 were identified, and in Google Scholar, 1650 papers were identified. After applying filters in Google Scholar (Thyroid crisis and propranolol and esmolol collapse storm or crisis arrest storm OR crisis "collapse"), 62 studies were the result, and 1588 were eliminated.

**Figure 1:**
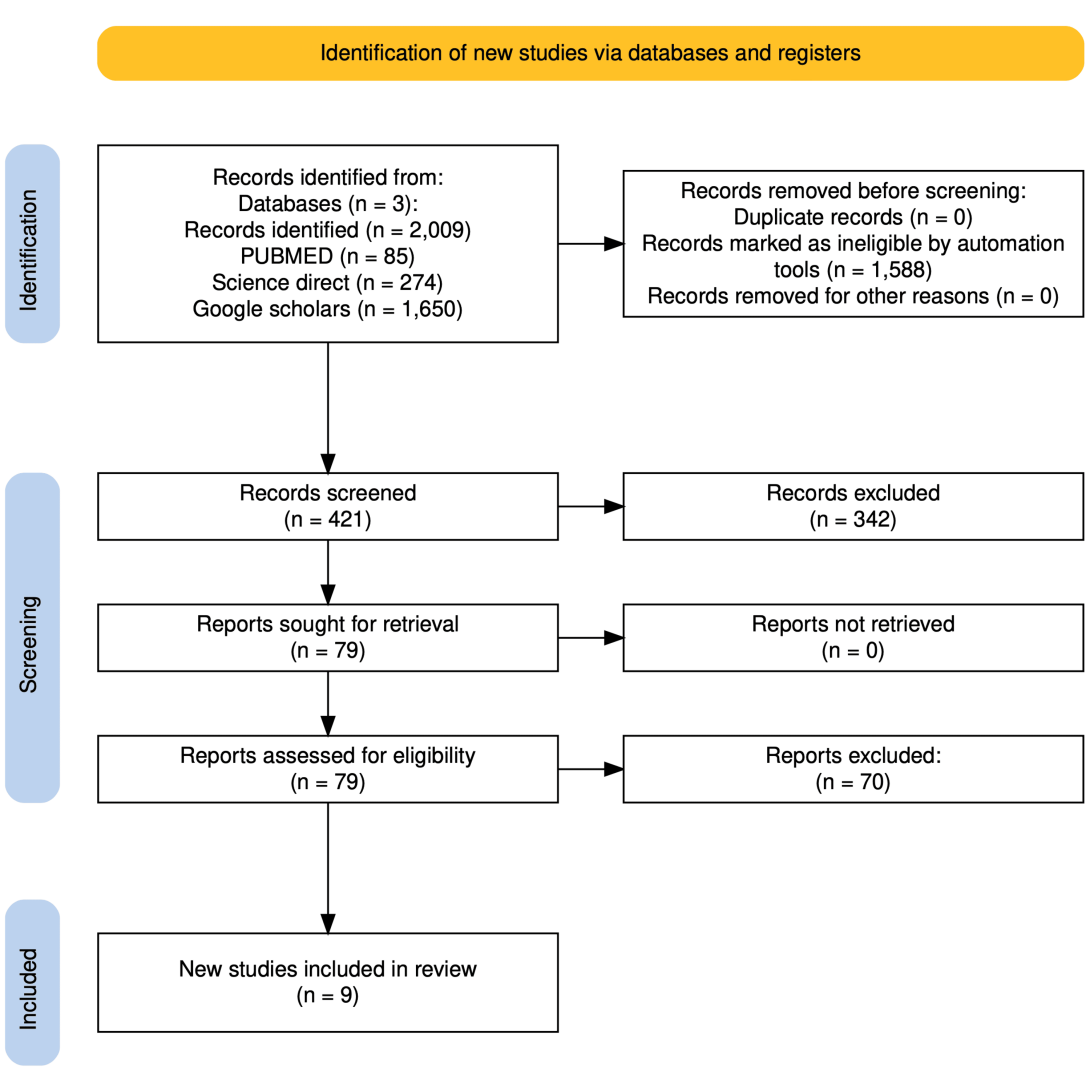
PRISMA flow chart PRISMA: Preferred Reporting Items for Systemic Reviews and Meta-Analysis

Four hundred twenty-one papers were shortlisted for screening. The papers were transferred manually to an Excel sheet (Microsoft Excel, Microsoft® Corp., Redmond, WA) and screened by reading the title and abstract and removing duplicates; 342 were taken out. Seventy articles were excluded after applying the inclusion and exclusion criteria due to either old-dated, concerning paediatric cases, or reporting complications other than cardiovascular collapse following beta-blocker administration. Finally, nine papers were shortlisted for this systemic review (Figure 1: PRISMA flow chart).

Discussion

A thyrotoxic storm is a rare, fatal medical emergency with a mortality rate of 10-30%. It can be the first presentation of thyroid disease or a complication of treatment non-compliance in known gravely ill individuals. Patients can have other medical issues, particularly cardiovascular disease, or be completely healthy and fit [3].

Presentation, Pathophysiology and Management 

Thyroid storms represent a state of extreme hyperthyroidism in which the thyroid gland synthesizes large amounts of thyroid hormones, leading to the severe systemic manifestations of thyrotoxicosis. The clinical picture can be like a hyperthyroid state or other systemic clinical signs and symptoms of neurological, cardiovascular, gastrointestinal, or thermoregulatory dysfunctions [4]. Most patients present with fever and cardiac features like tachycardia, heart failure, and arrhythmia. Nervous system symptoms include agitation, delirium, anxiety, psychosis, or coma. Gastrointestinal symptoms include nausea, vomiting, diarrhoea, abdominal pain, or acute liver failure. Additionally, multi-organ failure can be the acute presentation in the form of acute kidney injury (AKI), disseminated intravascular coagulation (DIC), or circulatory collapse that compromises the patients' prognosis [6].

Although the diagnosis of a thyrotoxic storm is clinical, laboratory investigations (thyroid function test, renal function test, electrolytes, liver function test, coagulation profile, full blood count, and blood glucose) and imaging (ECG, chest X-ray, echo, abdominal ultrasound, and CT head) can aid in the diagnosis. The Burch-Wartofsky Point Scale (BWPS) score indicates the likelihood of a thyroid crisis. A score of 45 and above shows a crisis; a score less than 25 is unlikely to be diagnosed; and a score in between is likely to be a crisis [6].

Treatment of thyroid storms consists of symptomatic treatment, circulatory support, and targeted therapy. Symptomatic as antipyretic, intravenous fluid, beta-blockers, or antiarrhythmic, circulatory support can be ECMO, plasma exchange, or plasmapheresis. Target therapy to either reduce hormonal production (ethionamide), stop the hormone release (iodine), or inhibit the peripheral conversion of T4 to T3 (PTU, beta-blockers, steroids, or radioactive iodine) [6].

Beta-Blockers Induced Circulatory Collapse

Beta-blockers have a vital role in treating thyrotoxicosis and TS. This group of medications plays several roles in managing arrhythmias, reducing hyperadrenergic states, and blocking peripheral T4 to T3 conversion [7]. On the other hand, like any other medication, it has adverse effects; they can range from dyspnoea, peripheral coldness, syncope, bradycardia, and hypotension to circulatory collapse and cardiac arrest [7]. Many literature reviews and case reports highlight propranolol-induced circulatory failure in patients with thyrotoxic crises. A very recent case series demonstrated that two young females aged 42 and 46 years with no past medical history of cardiac disease presented with TS. The treatment started with propranolol, steroids, and PTU. Shortly after beta-blocker intake, both went into cardiac arrhythmia and arrest [8].

Thyrotoxic cardiomyopathy was one of the theories behind the beta blocker-associated arrest, as shown in the case report of Cheah et al. [12], in which the patient was previously diagnosed with Graves’ disease, thyrotoxic cardiomyopathy, heart failure, and atrial fibrillation with an EF of 30%. Propranolol was given upon admission, and shortly afterwards, she went into pulseless electrical activity (PEA) arrest that was successfully resuscitated.

Management Before and After the Circulatory Collapse 

Almost all patients were transferred to the medical ICU unit following the cardiac event for close monitoring and extensive treatment. In most cases, beta-blockers, steroids, and anti-thyroid medications were the first line of treatment, followed by therapeutic plasma exchange, ECMO, and thyroidectomy in medical treatment failure cases.

Thyroidectomies were not performed in all patients, as in the study by Ali et al., where the patient recovered without needing the surgery and was discharged on medication [10]. Another example is the case report by Cheah et al., where the patient was treated with therapeutic plasma exchange and discharged with a plan for radioactive iodine therapy later [8].

Outcome

A substantial number of patients did not survive the beta blocker-triggered cardiac arrest despite all resuscitation and supportive measures, as in the study by Lim et al., where the patient suffered an irreversible hypoxic brain injury [9], and Moraco et al. blamed multi-organ failure [15].

On the other hand, many patients survived the TS and cardiac arrest, as in the systematic review by Modarresi et al., which showed that 15 out of 20 patients outlived the crisis and were discharged from the hospital in good condition [16].

Limitations

There were evident limitations to this paper. First, the number of cases available was scarce due to the condition's rarity; not many systematic reviews were available apart from the systemic review by Modarresi et al., and no RCTs were found. Furthermore, because of the unfavourable outcome in most scenarios, as in Lim et al. [9], Abubakar et al. [14], and Moraco et al. [15] case reports, not all cases were reported.

Second, the severity of the clinical presentation and its hideous course and complications limited the clinical trial establishment; hence, no consensus or universal treatment guidelines were available [3].

## Conclusions

The thyrotoxic crisis is a rare yet fatal thyroid disorder with a good prognosis if detected and treated promptly and effectively. Long-acting beta blockers, e.g., propranolol, were found to have a causal association with circulatory collapse and cardiac arrest in thyroid storm patients. Short-acting beta-blockers like Esmolol can play a pivotal role in thyrotoxic storm management, particularly in preventing arrhythmias and circulatory failure. This systematic literature review article demonstrated the specific benefit of using short-acting BB and the consequences associated with the use of long-acting BB; however, more precise and elaborated guidelines are required to produce more safe and trusted guidance on treating and preventing thyroid storms, especially in patients with pre-existing high-risk thyroid disorders.
