# Correction: Comparing the Incidence of Propranolol and Esmolol-Related Cardiac Arrest in Patients With Thyroid Storm: A Systematic Literature Review

**DOI:** 10.7759/cureus.c340

**Published:** 2025-10-01

**Authors:** Shadin Afifi, Vineet Suryadevara, Yaman Habab, Alana Hutcheson, Binay K Panjiyar, Gershon G Davydov, Hiba Nashat, Sally Ghali, Safeera Khan

**Affiliations:** 1 Internal Medicine, California Institute of Behavioral Neurosciences and Psychology, Fairfield, USA; 2 Global Clinical Scholar Research Training (GCSRT), Post Graduate Medical Education (PGMEE) at Harvard Medical School, Boston, USA; 3 Internal Medicine, California Institute of Behavioral Neurosciences and Psychology, California, USA; 4 Research, California Institute of Behavioral Neurosciences and Psychology, Fairfield, USA; 5 Internal Medicine, Soroka University Medical Center, Beer Sheva, ISR

This article has been corrected to fix multiple typos in the PRISMA diagram.

